# Supplementary material for: Five-year clinical outcomes of scanning carbon-ion radiotherapy for prostate cancer
Source: PLoS One. 2024 Mar 8;19(3):e0290617. doi: 10.1371/journal.pone.0290617 (PMC10923478; doi:10.1371/journal.pone.0290617)
Supplement: Attachment — Submitted filename: Responce to Reviewers.docx [file pone.0290617.s002.docx]

Reviewer #1:

We thank the reviewer for evaluating our manuscript. According to the suggestion, we revised the manuscript as follows.

“1- Now according to current standards of care and guidelines (NCCN), prophylactic nodal radiation is recommended for high-risk prostate cancer patients. It is suggested that the authors briefly discuss this issue/limitation in the discussion section, particularly in relation to carbon-ion therapy.”

Thank you for your precise comments. We had added the sentence in Materials and Methods section to explain that prophylactic pelvic irradiation was not performed as follows:

“Prophylactic irradiation of the pelvic region was not performed as in previous studies [7-16].” (Lines 87 to 88)

“2- Additionally, the discussion section should briefly mention the overall current radiotherapy treatment approach for prostate cancer patients and that caution should be exercised when interpreting the data due to the high degree of heterogeneity between this treatment (carbon ion) and photon therapy (such as direct comparison of fractionation, dose constraints) in the current literature.”

Thank you for your precise comments. We had added the sentence in Discussion section to explain that it cannot be compared our study with previous studies using conventional X-rays as follows:

“Fifth, RT is one of the definitive treatments for prostate cancer, however, the results of conventional RT with X-rays and CIRT in this study cannot be directly compared because of differences in dose fractionation and dose constraints.” (Lines 264 to 267)

Reviewer #2:

We thank the reviewer for evaluating our manuscript. According to the suggestion, we revised the manuscript as follows.

“In Discussion section (lines 220-223) please specify that PSA bounce occurs also post-SBRT. Reference recommened: “Jiang NY, Dang AT, Yuan Y, Chu FI, Shabsovich D, King CR, Collins SP, Aghdam N, Suy S, Mantz CA, Miszczyk L, Napieralska A, Namysl-Kaletka A, Bagshaw H, Prionas N, Buyyounouski MK, Jackson WC, Spratt DE, Nickols NG, Steinberg ML, Kupelian PA, Kishan AU. Multi-Institutional Analysis of Prostate-Specific Antigen Kinetics After Stereotactic Body Radiation Therapy. Int J Radiat Oncol Biol Phys. 2019 Nov 1;105(3):628-636. doi: 10.1016/j.ijrobp.2019.06.2539.””

Thank you for your precise comments. We had added the sentence in Discussion section to explain that PSA bounce was also observed after SBRT as follows:

“PSA bounce has also been reported after stereotactic body radiotherapy for prostate cancer [21].” (Lines 224 to 225)

And we have added the reference #21 as your recommendation in Lines 362 to 364
